# Alleviating chromium-induced oxidative stress in *Vigna radiata* through exogenous trehalose application: insights into growth, photosynthetic efficiency, mineral nutrient uptake, and reactive oxygen species scavenging enzyme activity enhancement

**DOI:** 10.1186/s12870-024-05152-y

**Published:** 2024-05-27

**Authors:** Amr Elkelish, Abdulrahman M. Alhudhaibi, ABM Sharif Hossain, Faouzi Haouala, Basmah M. Alharbi, Mostafa F. El-Banna, Amira Rizk, Arfang Badji, Nada Ibrahim AlJwaizea, Ali A. S. Sayed

**Affiliations:** 1 Department of Biology, College of Science, Imam Mohammad Ibn Saud Islamic University (IMSIU), Riyadh, 11623 Saudi Arabia; 2 Botany and Microbiology Department, Faculty of Science, Suez Canal University, Ismailia, 41522 Egypt; 3 Biology Department, Faculty of Science, University of Tabuk, Tabuk, 71491 Saudi Arabia; 4 Agricultural Botany Department, Faculty of Agriculture, Mansoura University, Mansoura, 35516 Egypt; 5 Department, Faculty of Agriculture, Tanta University, Tanta City, 31527 Egypt; 6 Department of Agricultural Production, College of Agricultural and Environmental Studies, Makerere University, P.O. Box 7062, Kampala, Uganda; 7 Department of Biology, College of science, Princess Nourah bint Abdulrahman University, P.O.Box 84428, Riyadh, 11671 Saudi Arabia; 8 Botany Department, Faculty of Agriculture, Fayoum University, Fayoum, 63514 Egypt; 9 Makerere University Regional Centre for Crop Improvement, Makerere University, Kampala, 7062, Uganda; 10 Biodiversity Genomics Unit, Faculty of Science, University of Tabuk, Tabuk, 71491 Saudi Arabia

**Keywords:** Trehalose, Oxidative damage, Antioxidants, Osmolytes, Photosynthesis, *Vigna radiata*

## Abstract

Trehalose serves as a crucial osmolyte and plays a significant role in stress tolerance. The influence of exogenously added trehalose (1 and 5 mM) in alleviating the chromium (Cr; 0.5 mM) stress-induced decline in growth, photosynthesis, mineral uptake, antioxidant system and nitrate reductase activity in *Vigna radiata* was studied. Chromium (Cr) significantly declined shoot height (39.33%), shoot fresh weight (35.54%), shoot dry weight (36.79%), total chlorophylls (50.70%), carotenoids (29.96%), photosynthesis (33.97%), net intercellular CO_2_ (26.86%), transpiration rate (36.77%), the content of N (35.04%), P (35.77%), K (31.33%), S (23.91%), Mg (32.74%), and Ca (29.67%). However, the application of trehalose considerably alleviated the decline. Application of trehalose at both concentrations significantly reduced hydrogen peroxide accumulation, lipid peroxidation and electrolyte leakage, which were increased due to Cr stress. Application of trehalose significantly mitigated the Cr-induced oxidative damage by up-regulating the activity of reactive oxygen species (ROS) scavenging enzymes, including superoxide dismutase (182.03%), catalase (125.40%), ascorbate peroxidase (72.86%), and glutathione reductase (68.39%). Besides this, applied trehalose proved effective in enhancing ascorbate (24.29%) and reducing glutathione content (34.40%). In addition, also alleviated the decline in ascorbate by Cr stress to significant levels. The activity of nitrate reductase enhanced significantly (28.52%) due to trehalose activity and declined due to Cr stress (34.15%). Exogenous application of trehalose significantly improved the content of osmolytes, including proline, glycine betaine, sugars and total phenols under normal and Cr stress conditions. Furthermore, Trehalose significantly increased the content of key mineral elements and alleviated the decline induced by Cr to considerable levels.

## Introduction

Heavy metal contamination has become a serious problem worldwide and has resulted in a significant threat to sustainable food production. The accumulation of heavy metals and metalloids in soils significantly affects plant growth, development, and productivity by influencing cellular division, photosynthesis, enzyme activity, and tolerance mechanisms [1, 2]. Chromium (Cr) is a toxic metal that potentially affects crop growth and development worldwide [3, 4]. It has been reported that Cr drastically reduces growth and development by affecting photosynthesis, mineral assimilation, and redox homeostasis reflecting an insignificant decline in [5]. Among the key symptoms of Cr toxicity are included: delayed seed germination, reduced biomass production, leaf chlorosis and photosynthetic arrest, altered nutrient uptake and assimilation, restricted water uptake and tissue proliferation and enzyme inhibition ultimately affecting the yield potential of the plant [6–9]. Wheat plants treated with Cr exhibit significant alteration in photosynthetic attributes like the number of active PSII reaction centers, electron transport and activity of PSII heterogeneity [10]. Cr exists as Cr(III) and Cr(VI) valence states in which Cr(VI) is more toxic and persistent in soil, and has higher solubility and mobility in water [11, 12]. Plants take Cr from the soil through phosphate and sulphate transporters [12]. Among the natural sources of Cr accumulation mineral leaching is the prime contributor while the anthropogenic sources contributing 70% of the total Cr accumulation in soil include the effluent from paper and pulp mills, leather tanning industries, non-ferrous base metal smelters, refineries, thermal generating stations and the urban stormwater runoff [12–15].

Once taken up by plants Cr triggers toxic damaging effects by inducing the formation of reactive oxygen species thereby hampering normal growth and metabolism in plants by interfering with pigment synthesis, photosynthesis, root growth and osmolyte metabolism [16]. Plants have naturally occurring tolerance mechanisms to mitigate the negative effects of stresses to safeguard the metabolism and other machinery for better growth and yield performance [17, 18]. The prime mechanisms and pathways contributing to stress tolerance in plants include antioxidant system, osmolyte accumulation and efficient exclusion and sequestration of toxic ions [18–21]. Plants exhibiting significant up-regulation of the tolerance mechanisms mitigate the damaging effects of stresses efficiently [22–25]. Several strategies including molecular interventions, the use of novel management strategies, exogenous supplementation of minerals, phytohormones and metabolites, etc. have been exploited to improve the tolerance mechanisms to strengthen the potentiality of plants to withstand adverse environmental conditions. For example, the exogenous application of nitric oxide [26] and acetylcholine [27] improved tolerance to mercury and salinity treatments through the strengthening of tolerance mechanisms. In wheat, Adrees et al. (2015) have demonstrated that the application of mannitol improves the functioning of the antioxidant system thereby reducing oxidative damage and the Cr uptake [28].

In addition to proline, glycine betaine, etc., trehalose is considered a key sugar osmolyte having a substantial role in imparting stress tolerance. It plays a role in the regulation of plant growth, development, and metabolism in response to extreme environments such as high temperature, salinity, drought, and cold stress [29]. Trehalose 6-phosphate (T6P) was considered to be a signaling metabolite communicating the carbohydrate state of plants to other pathways involved in growth, development and responses to the environment [30]. Therefore, the various metabolic functions of trehalose may be caused by its role in sugar signal transduction. It restricts the growth of pathogens and also elicits the tolerance to withstand stress conditions [31]. As a protectant under stressful conditions, trehalose alleviates stress-induced physiological and biochemical disorders, delays leaf abscission and stimulates flowering [32]. In rice treatment of trehalose has been reported to alleviate the damaging effects of cadmium [33] and salinity [34].

*Vigna radiata* is an important legume crop also known as green gram or mung bean and is grown worldwide for its edible seeds that are rich in carbohydrates, proteins and minerals. The nitrogen-fixing ability of green gram benefits to improve the soil fertility and hence contributes to betterment of plant growth. The excess accumulation of metals including Cr can be toxic to growth of *Vigna radiata* and hence can result in a significant decline in its yield. In this backdrop it was hypothesized that the exogenous application of trehalose can mitigate the negative effects of Cr on growth, photosynthesis and mineral uptake by up-regulating the antioxidant functioning and osmolyte accumulation.

## Materials and methods

### Plant Material

Seeds of *Vigna radiata* were surface sterilized and sown in earthen pots filled with sand and soil in a ratio of 3:1. At the time of sowing all pots were irrigated with 250 mL full-strength Hoagland nutrient solution. In this study, all chemicals used were of analytical grade. Specifically, trehalose was procured from Sigma-Aldrich, Germany. Five days after germination two healthy and uniformly growing seedlings were maintained in each pot and were irrigated with full-strength Hoagland solution on every alternate day for another five days. Ten days after normal seedling growth pots were divided into two groups and were irrigated with normal Hoagland and modified Hoagland solution containing 0.5 mM Cr (in the form of K_2_Cr_2_O_7_) to induce Cr stress. Treatment of trehalose (1 and 5 mM) was given foliarly using 0.1% tween-20 as surfactant and during the treatment of trehalose, pots were covered with polythene to avoid the penetration of trehalose into roots. Trehalose was given twice a week (15 mL per pot). Pots were arranged in a complete randomized block design with five replicates for each treatment and were kept in a greenhouse under natural conditions with day/ night temperatures (^o^C) of 35/26 ± 5 and relative humidity (percent) of 75/55 ± 8. Twenty days after Cr and trehalose treatment plant were uprooted carefully and analyzed for different parameters as described below.

### Estimation of total chlorophylls and carotenoids, and gas exchange parameters

The content of total chlorophylls and carotenoids was estimated according to [35] and optical density was taken at 480, 645 and 663 nm. LI-6400 photosynthesis system (Li-Cor, USA) was used for the estimation of net photosynthesis, intercellular CO_2_ and transpiration rate, and the observations were recorded in a fully expanded leaf between 09:00–12:00 h.

### Measurement of osmolytes and RWC

The extraction of soluble sugar was in ethanol and estimations were carried out according to the anthrone method [36]. For estimating proline content method of [37] was adopted and extraction was 3% sulphosalicylic acid. Optical density was taken at 520 nm. Glycine betaine was estimated according to [38] and absorbance was recorded at 365 nm. For relative water content (RWC) determination method of [39] was used. Leaf discs were punched from fresh leaf tissue and their fresh weight (FW) was taken and turgid weight (TW) of same leaf discs was taken after allowing them to gain turgidity in distilled water for 1 h. Dry weight (DW) of discs was recorded after oven drying the discs for 24 h at 80 °C. Calculation was carried using formula:

$$\text{R}\text{W}\text{C}\left(\text{\%}\right)=\frac{\text{F}\text{W}-\text{D}\text{W}}{\text{T}\text{W}-\text{D}\text{W}} \text{x} 100$$

### Lipid peroxidation, hydrogen peroxide and electrolyte leakage

For lipid peroxidation [40], method was followed and 100 mg fresh tissue was extracted in 1% TCA. The formation of malonaldehyde (MDA) content was recorded at 532 and 600 nm. Hydrogen peroxide was estimated by crushing 500 mg fresh tissue in 0.1% TCA and supernatant was mixed with potassium phosphate buffer (pH 7.0) and 1 mL potassium iodide solution. Absorbance was taken at 390 nm [41]. Electrolyte leakage was measured following [42].

### Assay of nitrate reductase

Nitrate reductase (E.C. 1.6.6.1.) activity was assayed following [43] and absorbance was recorded at 540 nm.

### Determination of antioxidant enzyme activities

Extraction of antioxidant enzymes was carried out by homogenizing 500 mg fresh tissue in sodium phosphate buffer (50 mM, pH 7.0) containing 1% PVP, 0.5 mM EDTA and 0.1 mM PMSF. After centrifuging at 15,000 g for 20 min at 4 °C, supernatant was used for enzyme assay. Thereafter the activity of superoxide dismutase [44], catalase [45], ascorbate peroxidase [46] and glutathione reductase [47] was measured. Protein determination method of [48] was used.

### Measurement of ascorbate and reduced glutathione

Ascorbate (AsA) was extracted in 6% TCA and supernatant was mixed with 2% dinitrophenylhydrazine and thiourea, and absorbance was taken at 530 nm [49]. Reduced glutathione was extracted by homogenizing fresh tissue in phosphate buffer (pH 8.0) and supernatant was mixed with 5, 5-dithiobis-2-nitrobenzoic acid and absorbance was taken at 412 nm [50].

### Estimation of total phenols

Total phenols were extracted in methanol and after centrifuging at 10,000 g for 10 min supernatant was reacted with Folin–Ciocalteu reagent. Optical density was measured at 765 nm [51].

### Estimation of nutrients and chromium

For the estimation of nitrogen method of [52] was adopted. The content of K, Ca and Mg were determined using atomic absorption Spectrophotometer (Shimadzu AA-6300, Japan) [53]. The spectrophotometric method described by [54] was used for the estimation of phosphorous. Sulphur was estimated using the turbidimetric method [55].

### Estimation of cr content in shoot & root is missing

Cr content in shoots and roots was quantified at 50 days after seeding? following the method of McGrath & Cunliffe [56] and Ouzounidou et al. [57]. Root and shoot samples (0.5 g each) were digested in 15 ml conc. HNO_3_:HClO_4_ (3:1, v/v) on a hot plate, by gradually increasing the temperature up to 275 °C till yellow fumes began to emerge from the flask. H_2_O_2_ was added once the density of fumes decreased. After cooling, distilled water was added to the samples to achieve a total volume of 25 ml. Cr content was determined by obtaining absorbance values using flame atomic absorption spectrophotometry.

### Statistical analysis

The obtained data were analyzed using one-way analysis of variance (ANOVA), using Minitab® Statistical Software version 20, and the results were described as a mean of three replicates ± standard error [58]. The least significant was calculated using Duncan’s Multiple Range Test at *p* < 0.05.

## Results

Cr stress significantly reduced shoot height, fresh weight, and dry weight of the shoot. However, trehalose application not only increased these parameters but also alleviated the decline induced by Cr. Relative to control, treatment of Cr declined shoot height by 39.33%, shoot fresh weight by 35.54% and shoot dry weight by 36.79% (Table 1). Application of trehalose at both concentrations alleviated the decline to significant levels (Table 1). Treatment of Cr declined the content of N, P, K, S, Mg and Ca by 35.04%, 35.77%, 31.33%, 23.91%, 32.74% and 29.67% respectively as compared to control. Application of trehalose mitigated to decline to considerable levels with maximal mitigation due to 5 mM trehalose. Percent decline in Cr + 5 mM trehalose-treated plants was 1.02%, 2.11%, 2.96%, 1.49%, 2.29% and 7.64% for N, P, K, S, Mg and Ca respectively over control (Table 1). Under normal conditions, both concentrations of trehalose enhanced N, P, K, S, Mg and Ca maximally by 26.27%, 31.69%, 44.52%, 60.89%, 33.58% and 42.90% respectively due to 5 mM trehalose (Table 1). Relative to control, Cr content in the shoot decreased by 17.41% and 60.78% due to the application of 1- and 5-mM trehalose over the Cr-stressed counterparts (Table 1). A more or less similar impact was observed in the root (Table 1).

**Table 1:** Effect of chromium stress on shoot length, shoot fresh weight, shoot dry weight, content of nitrogen, phosphorous, potassium, sulphur, magnesium, calcium and chromium in *Vigna radiata* plants grown with and without exogenous application of trehalose (Tre; 1 and 5 mM). Data is mean (± SE) of three replicates and means followed by same letters are not significantly different at *p* < 0.05

	Control	1 mM Tre	5 mM Tre	Cr	Cr + 1 mM Tre	Cr + 5 mM Tre
Shoot length	24.76 ± 2.4c	27.01 ± 2.7b	33.87 ± 3.2a	15.02 ± 1.13f	17.91 ± 1.51e	20.21 ± 2.1d
Shoot fresh weight	4.98 ± 0.22c	5.44 ± 0.27b	7.25 ± 0.52a	3.21 ± 0.17ef	3.78 ± 0.29e	4.24 ± 0.32d
Shoot dry weight	2.31 ± 0.16c	2.79 ± 0.18b	3.31 ± 0.18a	1.46 ± 0.091f	1.69 ± 0.092e	1.97 ± 0.089d
Nitrogen	30.1 ± 2.5c	34.2 ± 2.9b	38.01 ± 3.3a	19.55 ± 1.7f	24.99 ± 1.9e	29.79 ± 2.1d
Phosphorous	14.2 ± 1.01c	15.9 ± 1.11b	18.7 ± 1.52a	9.12 ± 0.82ef	10.8 ± 0.88e	13.9 ± 1.01d
Potassium	17.54 ± 1.3c	21.11 ± 2.1b	25.35 ± 2.6a	12.07 ± 0.78f	14.43 ± 1.10e	17.02 ± 1.72d
Sulphur	8.03 ± 0.62c	9.31 ± 0.88b	12.92 ± 1.01a	6.11 ± 0.42ef	6.83 ± 0.301e	7.91 ± 0.41d
Magnesium	13.1 ± 1.01c	14.8 ± 1.13b	17.5 ± 1.35a	8.81 ± 0.56ef	9.3 ± 0.73e	12.8 ± 0.938d
Calcium	11.12 ± 1.13c	13.09 ± 1.19b	15.78 ± 1.31a	7.82 ± 0.67f	8.84 ± 0.72e	10.27 ± 0.889d
Shoot Chromium	ND	ND ±	ND	29.45 ± 2.6a	24.32 ± 2.4b	11.55 ± 0.82c
Root Chromium	0.2112 ± 0.009d	0.2019 ± 0.0088d	0.2102 ± 0.011d	36.21 ± 3.8a	29.44 ± 3.1b	17.57 ± 1.26c

**Fig. 1:**
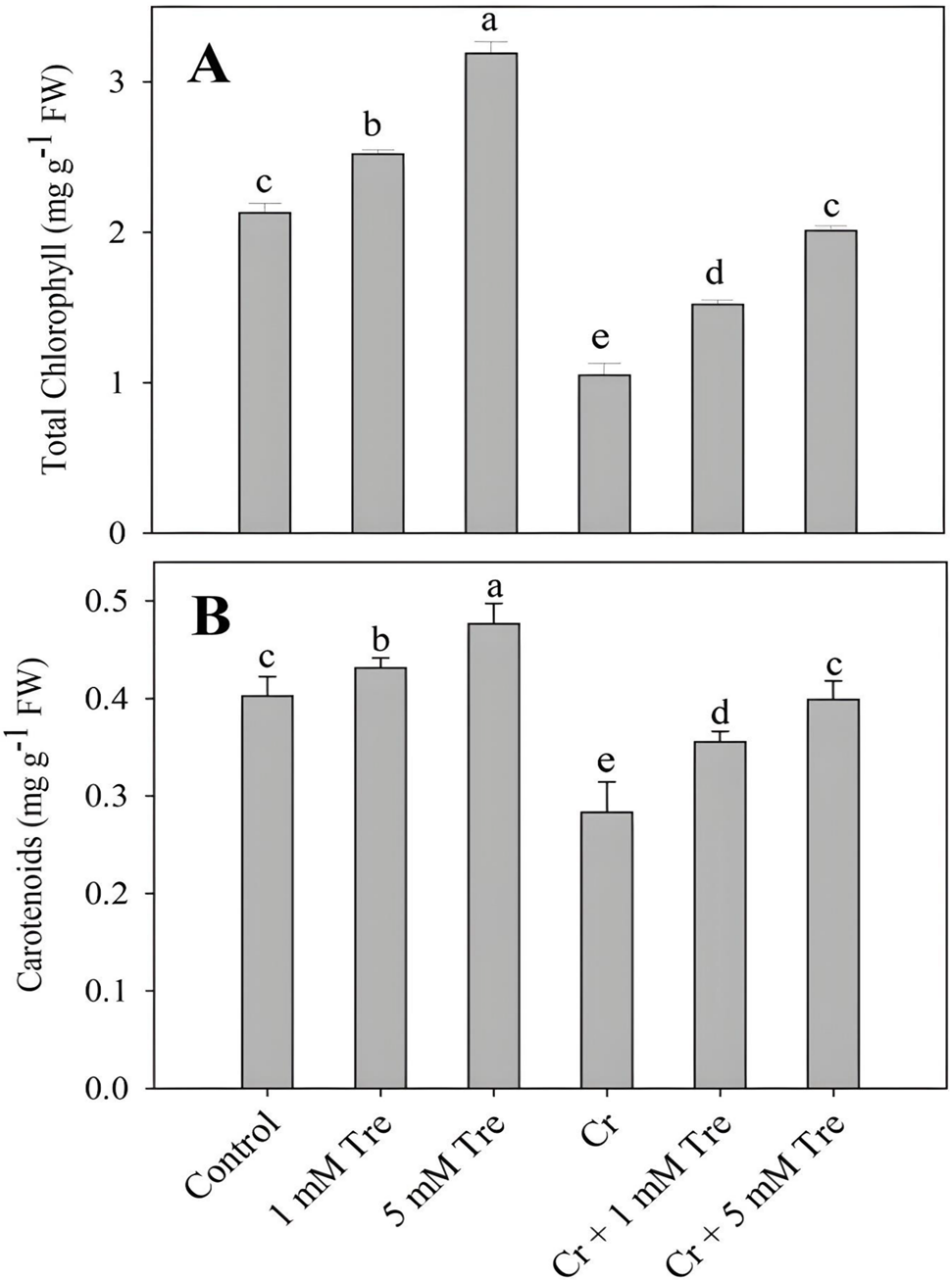
Effect of chromium stress on (**A**) Total chlorophyll and (**B**) carotenoids in *Vigna radiata* plants grown with and without exogenous application of trehalose (Tre; 1 and 5 mM). Data is mean (± SE) of three replicates and means followed by same letters are not significantly different at *p* < 0.05

**Fig. 2:**
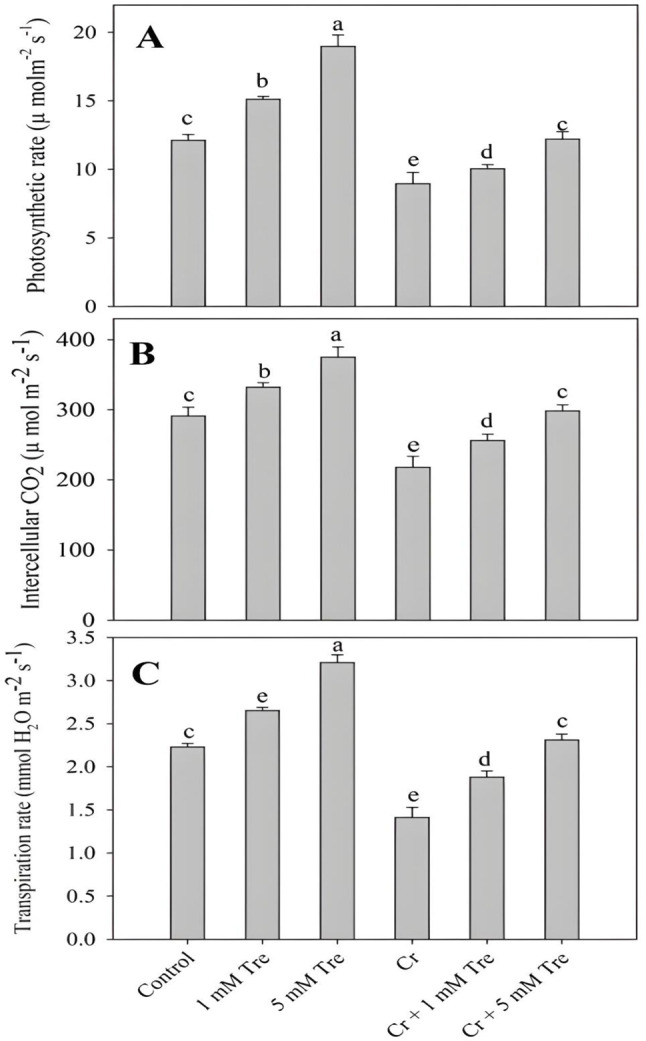
Effect of chromium stress on (**A**) Net photosynthesis, (**B**) intercellular CO_2_ concentration and (**C**) stomatal conductance in *Vigna radiata* plants grown with and without exogenous application of trehalose (Tre; 1 and 5 mM). Data is mean (± SE) of three replicates and means followed by same letters are not significantly different at *p* < 0.05

Supplementation of trehalose significantly increased total chlorophylls, carotenoids, photosynthesis, net intercellular CO_2_ and transpiration rate with maximal increases of 49.76%, 18.39%, 56.68%, 42.06% and 43.94% observed due to 5 mM concentration of trehalose. Treatment of Cr resulted in a decline of 50.70% in total chlorophylls, 29.96% in carotenoids, 33.97% in photosynthesis, 26.86% in net intercellular CO_2_ and 36.77% in transpiration rate over the control. Application of trehalose to Cr-stressed plants considerably mitigated Cr-induced decline in total chlorophylls, carotenoids, photosynthesis, net intercellular CO_2_ and transpiration rate with maximal alleviation observed due to 5 mM trehalose (Figs. 1 and 2).

**Fig. 3:**
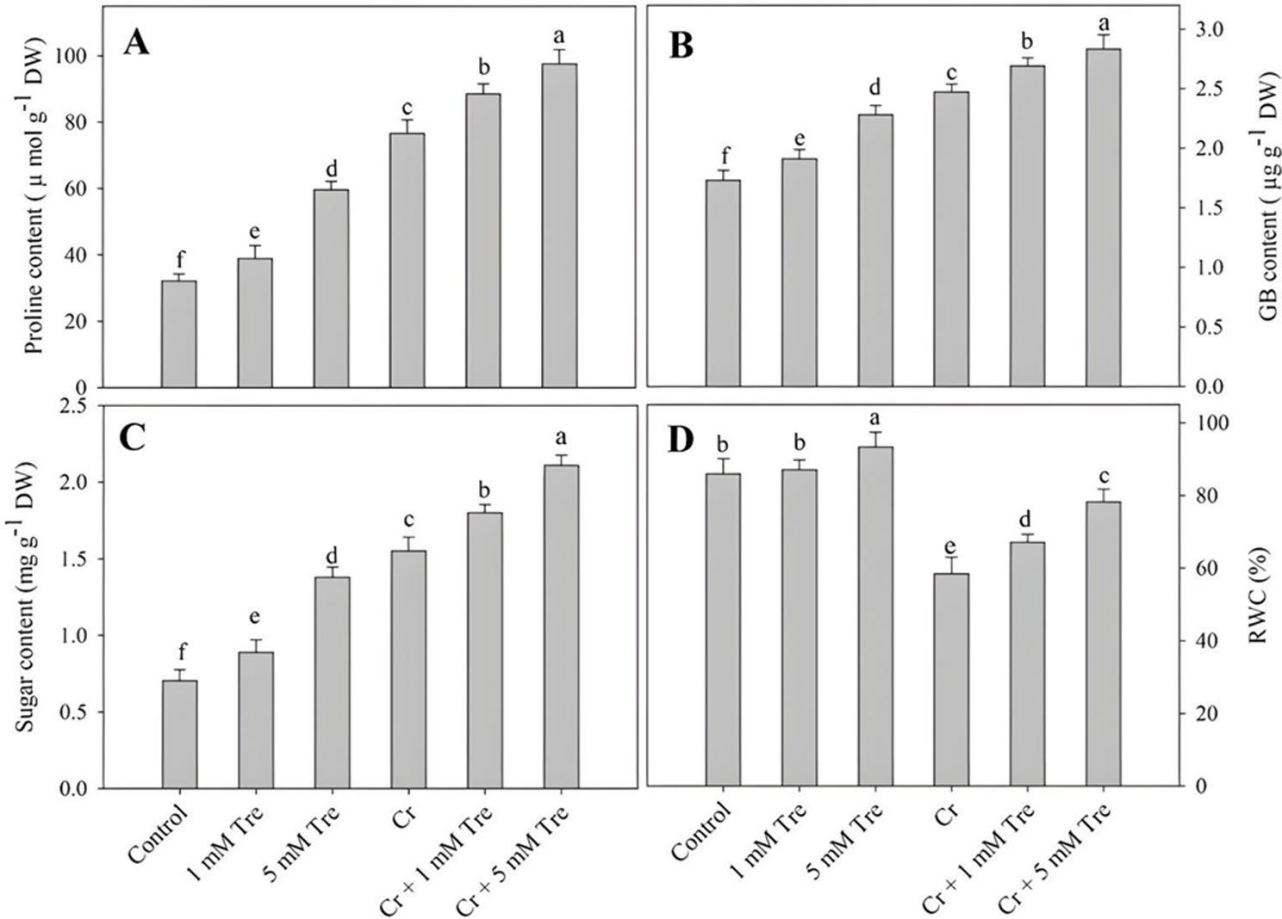
Effect of chromium stress on (**A**) proline, (**B**) glycine betaine, (**C**) sugar and (**D**) relative water content (RWC) in *Vigna radiata* plants grown with and without exogenous application of trehalose (Tre; 1 and 5 mM). Data is mean (± SE) of three replicates and means followed by same letters are not significantly different at *p* < 0.05

Treatment of Cr increased the accumulation of sugars, proline and glycine betaine. Application of trehalose at 1 mM concentrations increased the content of sugars proline and glycine betaine by 26.45%, 20.97% and 10.40% respectively while as 5% trehalose caused an increase of 96.30%, 85.34% and 31.79% respectively. Application of trehalose to Cr-treated plants enhanced the sugars, proline and glycine betaine. Sugars, proline and glycine betaine maximally increased by 200.13%, 203.42% and 63.58% respectively in Cr + 5 mM trehalose-treated plants (Fig. 3A-C). Relative to control, RWC was decreased by 32.00% due to Cr stress and increased by 8.62% due to 5 mM trehalose treatment. The decline in RWC was alleviated by the supplementation of trehalose (Fig. 3D).

**Fig. 4:**
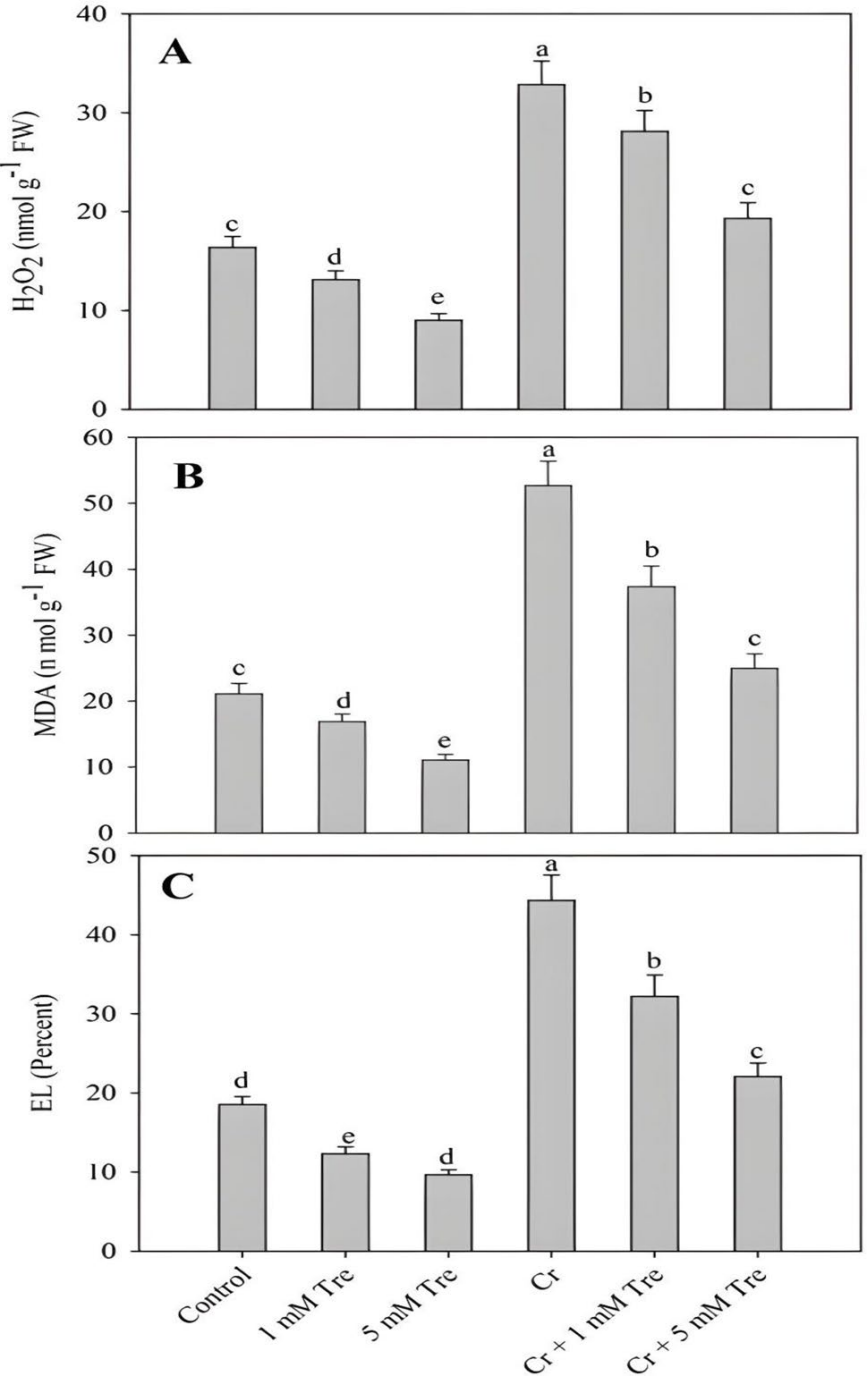
Effect of chromium stress on (**A**) hydrogen peroxide, (**B**) Lipid peroxidation and (**C**) electrolyte leakage in *Vigna radiata* plants grown with and without exogenous application of trehalose (Tre; 1 and 5 mM). Data is mean (± SE) of three replicates and means followed by same letters are not significantly different at *p* < 0.05

Cr stress increased the generation of H_2_O_2_, lipid peroxidation and electrolyte leakage significantly over control, however, the application of trehalose reduced H_2_O_2_, lipid peroxidation and electrolyte leakage under normal as well as Cr treatments. Relative to control, H_2_O_2_, lipid peroxidation and electrolyte leakage increased by 131.09%, 149.85% and 139.23% respectively due to Cr stress. Treatment of 5 mM trehalose reduced the H_2_O_2_, lipid peroxidation and electrolyte leakage by 44.96%, 47.43% and 48.08% respectively as compared to control. Application of trehalose to Cr-stressed plants alleviated the H_2_O_2_, lipid peroxidation and electrolyte leakage with maximal alleviation observed in Cr + 5 mM trehalose-treated plants (Fig. 4A-C).

**Fig. 5:**
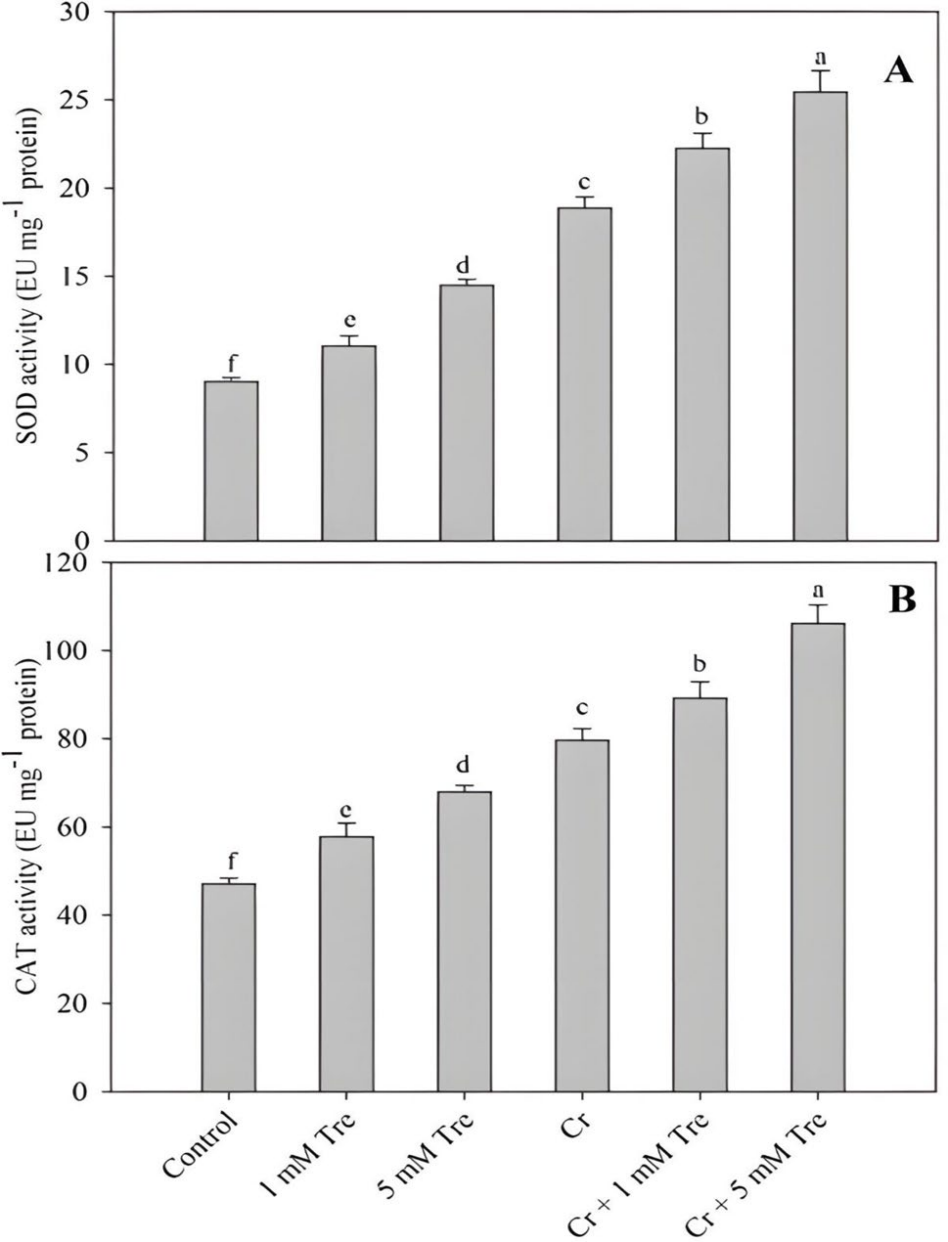
Effect of chromium stress on the activity of (**A**) superoxide dismutase and (**B**) catalase in *Vigna radiata* plants grown with and without exogenous application of trehalose (Tre; 1 and 5 mM). Data is mean (± SE) of three replicates and means followed by same letters are not significantly different at *p* < 0.05

**Fig. 6:**
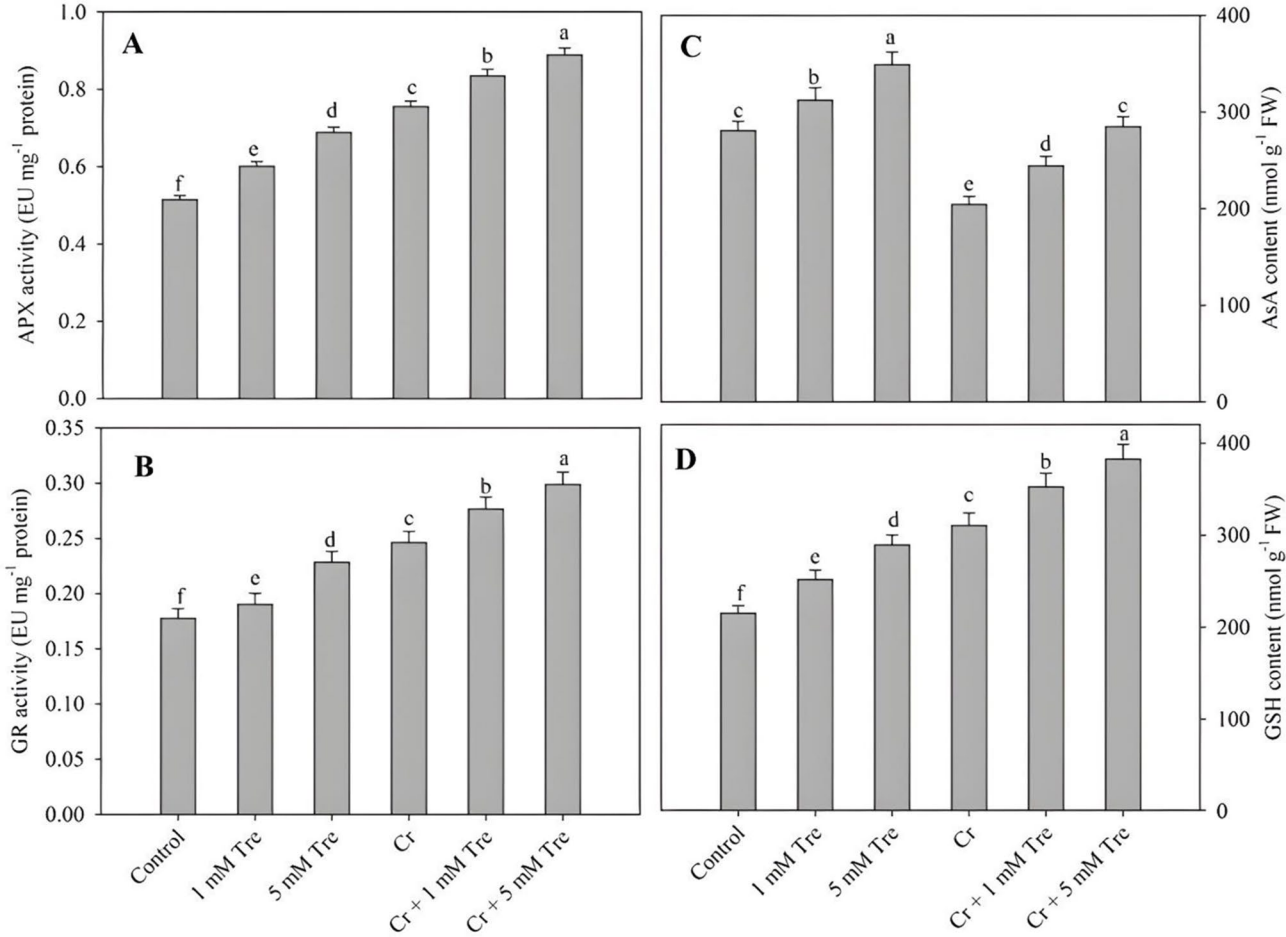
Effect of chromium stress on the activity of (**A**) ascorbate peroxidase, (**B**) glutathione reductase and content of (**C**) ascorbate and (**D**) reduced glutathione in *Vigna radiata* plants grown with and without exogenous application of trehalose (Tre; 1 and 5 mM). Data is mean (± SE) of three replicates and means followed by same letters are not significantly different at *p* < 0.05

The activity of SOD, CAT, APX and GR increased by 60.42%, 44.20%, 33.78% and 28.56% respectively due to the application of 5 mM trehalose. In Cr-stressed plants activity of SOD, CAT, APX and GR increased by 108.98%, 69.15%, 46.79% and 38.64% respectively, however, plants treated with Cr + 5mM trehalose resulted in an increase of 182.03%, 125.40%, 72.86% and 68.39% in SOD, CAT, APX and GR over the control (Figs. 5A and B and 6A and B). The content of AsA decreased by 27.25% and GSH increased by 44.35% due to Cr stress compared to control. Under normal growth conditions, the application of trehalose maximally increased AsA and GSH by 24.29% and 34.40% at 5 mM concentrations. Treatment of trehalose to Cr-stressed plants alleviated the decline in AsA while causing a further increase in GSH content (Fig. 6C and D).

**Fig. 7:**
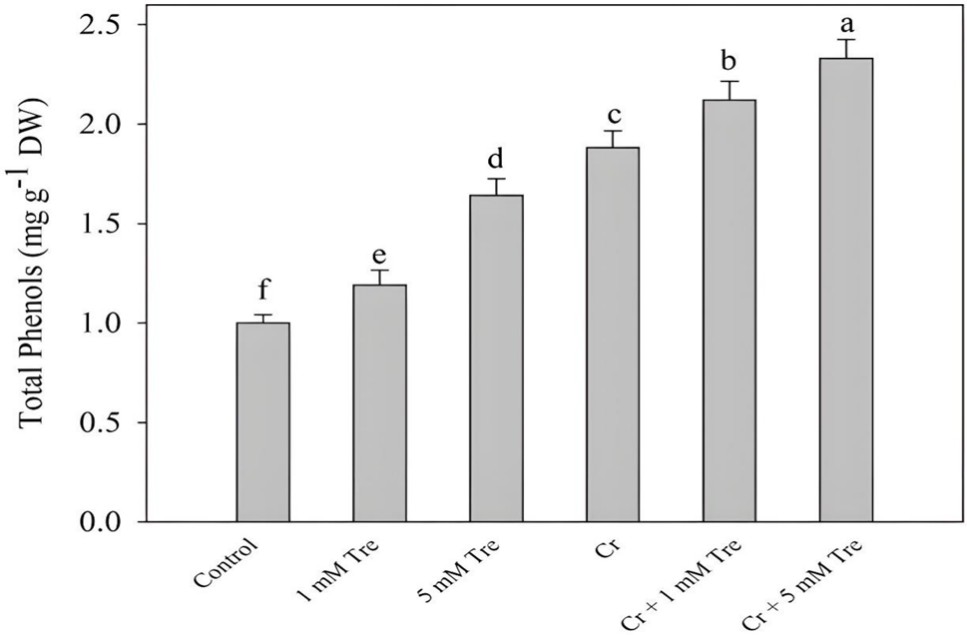
Effect of chromium stress on total phenol content in *Vigna radiata* plants grown with and without exogenous application of trehalose (Tre; 1 and 5 mM). Data is mean (± SE) of three replicates and means followed by same letters are not significantly different at *p* < 0.05

The content of total phenols increased due to the application of trehalose by 19.00% and 64.00% due to 1- and 5-mM concentrations. Seedlings treated with Cr exhibited an increase of 88.00% in total phenols which was further increased due to the application of trehalose. Maximum accumulation of total phenols was observed in plants treated with Cr and 5 mM trehalose attaining an increase of 133.00% over control (Fig. 7).

**Fig. 8:**
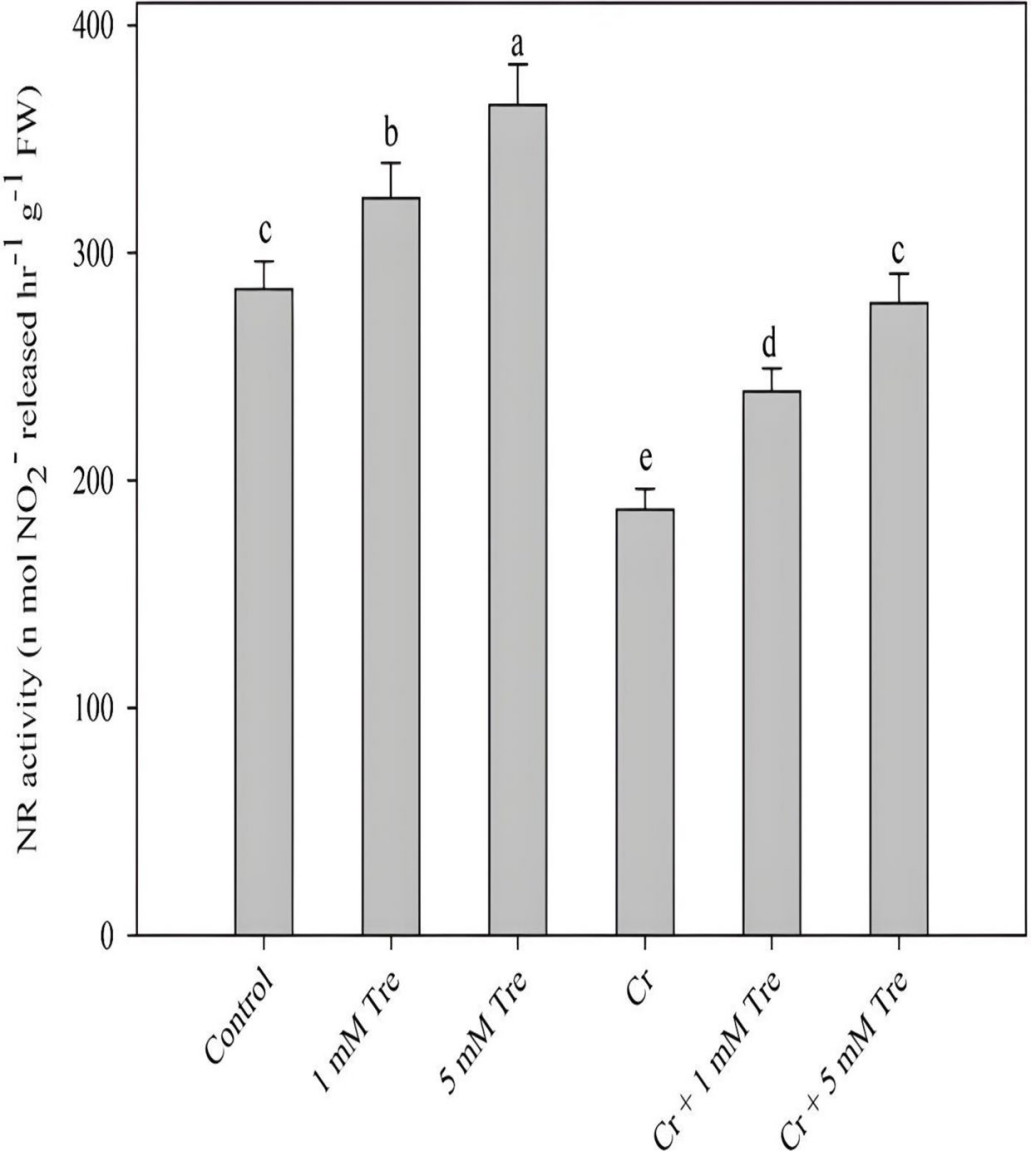
Effect of chromium stress on activity of nitrate reductase in *Vigna radiata* plants grown with and without exogenous application of trehalose (Tre; 1 and 5 mM). Data is mean (± SE) of three replicates and means followed by same letters are not significantly different at *p* < 0.05

Cr stress significantly reduced the nitrate reductase activity and the application of trehalose not only increased its activity but also alleviated the decline induced by Cr. Percent decline in activity due to Cr was 34.15% while as trehalose increased the activity by 14.08% and 28.52% at 1 and 5 mM respectively compared to control. The decline in Cr + 5 mM trehalose-treated plants was 2.11% over control (Fig. 8).

## Discussion

Rapid industrialization has a significant contribution to increasing metal pollution all around the globe. This has led to a considerable decline in the arable productive land thereby significantly reducing the yield. To protect growth and yield production from the adverse effects of toxic metals different management strategies have been devised and implemented from time to time. The present study aimed to evaluate the potentiality of applied trehalose in the mitigation of Cr toxicity-induced growth and metabolic alterations in *Vigna radiata*. Treatment of trehalose at 1 and 5 mM proved beneficial in alleviating the Cr-mediated growth reduction to some extent. Earlier it was been reported that Cr stress reduced growth, fresh and dry weight significantly in different plants [5, 9]. Cr potentially reduces the germination, and growth of coleoptile, hinders root elongation and distorts the xylem and phloem [59]. In *Vigna radiata*, treatment of Cr significantly reduces growth and yield by triggering oxidative damage [4]. Adverse effects of cadmium in rice [33], salinity in wheat [60] and drought in sweet basil [61] on the growth, fresh and dry biomass were mitigated by trehalose. Trehalose application potentially enhances ABA to induce salt tolerance in tomato [62]. Transgenic rice exhibiting up-regulated trehalose biosynthesis depicts better growth, increased photosynthesis, Na/K and relative water content under drought, salinity and sodic stress [63]. Increased growth in trehalose-treated plants may be attributed to significant enhancement in essential mineral ions, increased chlorophyll synthesis and osmolyte accumulation in addition to up-regulated antioxidant system. Trehalose application resulted in significant improvement in the content of key elements including N, P, K, S, Mg and Ca thereby assisting the plants to maintain metabolic functioning and growth under Cr stress conditions. Optimal availability of N [64, 65], P [19, 20], K [66, 67], S [68], Mg [69] and Ca [18] enhance growth and improve stress tolerance by regulating key functions involving enzyme activity, chlorophyll synthesis, mineral uptake and assimilation, protein synthesis, osmolyte accumulation and antioxidant functioning [70]. have also demonstrated a significant decline in the content of K, Mg, Fe and Zn in Cr-stressed lettuce. In the present study trehalose application resulted in a significant enhancement in the uptake of essential mineral elements and reduced the accumulation of Cr thereby contributing to growth improvement and Cr tolerance. Magnesium is a key central component of chlorophyll and trehalose-mediated enhancement in Mg may have contributed significantly to chlorophyll synthesis thereby contributing to increased growth and photoassimilate production. Cr impairs the nutrient balance by reducing their assimilation [71] and a significant reduction in key mineral ions has been reported in paddy under Cr stress [72]. Besides, Cr imparted an obvious decline in the activity of nitrate reductase reflecting in its negative influence on the assimilation of nutrients as well. Earlier Cr-induced reduction in nitrate reductase activity has been reported which severely influences the photosynthetic efficiency [5]. Decline nitrate reductase activity due to Cr stress is mainly due to impaired substrate utilization as has been reported by [73]. Trehalose application may have contributed to improved nutrient uptake by regulating the activity of mineral transport proteins and preventing the inhibitory effects of Cr on the assimilatory pathways. Besides this increased root growth and root penetration as well as improved translocation of nutrients due to trehalose application may have served as a key factor for increased mineral uptake.

Cr stress reduced the chlorophyll and carotenoid content as well as photosynthesis, CO_2_ concentration and transpiration rate. On the contrary, plants treated with trehalose exhibited a significant increase in these parameters and trehalose was effective in mitigating the Cr-induced decline. Earlier [5], reported a significant decline in chlorophyll, net photosynthesis, stomatal conductance, intercellular CO_2_ concentration, electron transport rate, PSII functioning and Rubisco activity in Cr-stressed Brassica juncea plants. Cr affects chloroplast morphology and volume besides obvious influence on pigment synthesis and photosynthetic gas exchange parameters in pea [74]. Reduced chlorophyll synthesis, photosynthesis, *Fv/Fm* and electron transport rate due to Cr stress is attributed to increased generation of toxic free radicals like superoxide, hydrogen peroxide and hydroxyl reflecting in declined growth and biomass production [75]. Exogenous trehalose supplementation has been reported to increase chlorophyll [34] and carotenoid [76] content in *Oryza sativa* and *Raphanus sativus* and alleviate the decline induced due to salinity and water deficit stresses respectively. Trehalose supplementation improves photosynthetic carbon assimilation under heat stress in wheat and maize [77] [61]. also demonstrated increased net photosynthesis, intercellular CO_2_, transpiration rate and stomatal conductance concomitant with significant alleviation of drought-induced decline due to the application of trehalose. Stresses induce ROS production thereby hampering the structural and functional integrity of chloroplast and also up-regulate the functioning of chlorophyll degrading enzymes with a significant decline in the activity of those involved in biosynthesis [27]. Trehalose-treated plants exhibited lesser accumulation of ROS concomitant with increased water content and mineral (N and Mg) content which may have contributed to improved chlorophyll synthesis and photosynthesis in them. Carotenoids contribute to ROS scavenging besides their key role in photoprotection [78] and trehalose-induced increase in carotenoids resulted in improved growth and stress tolerance by improving photosynthesis and photoprotection, phytohormone synthesis and signaling. Plants exhibiting increased synthesis of carotenoids have improved potential to withstand stress [79].

Reduced growth, chlorophyll synthesis and photosynthesis in Cr-stressed plants were correlated with significant enhancement in the oxidative damage measured in terms of H_2_O_2_, lipid peroxidation and electrolyte leakage. Increased ROS accumulation and membrane damage in terms of lipid peroxidation and electrolyte leakage are in corroboration with [5, 80, 81] and [4]. Stress conditions trigger the peroxidation of membrane lipids and up-regulate lipoxygenase activity resulting in a significant decline in the integrity of membranes [22, 64, 82] hence functioning of key cellular compartments is impeded. In Cr-stressed *Cicer arietinum* L [9], has also demonstrated increased H_2_O_2_ content, lipid peroxidation and electrolyte leakage resulting in significant growth and yield restriction. Oxidative damage triggered by Cr was alleviated by the application of trehalose at both concentrations with 5 mM to be much more effective. The application of trehalose has been reported to alleviate the damaging effects of salinity stress on growth and photosynthesis by reducing the accumulation of H_2_O_2_, lipid peroxidation and lipoxygenase activity in wheat [60]. Excess accumulation of ROS damages cellular structures, hampers photosynthesis and weakens the tolerance mechanisms however exogenous trehalose application prevents the adverse effects to significant levels [61]. The trehalose-mediated decline in ROS, lipid peroxidation, electrolyte leakage and lipoxygenase is reflected in significant protection of proteins in thylakoid membranes and photosynthesis [83]. Trehalose application resulted in a significant enhancement in membrane stability (obvious as reduced oxidative damage) reflecting in increased growth and photosynthesis.

Reduced oxidative damage in trehalose-treated plants can be ascribed to improved functioning of tolerance mechanisms including antioxidant system, and accumulation of osmolyte and secondary metabolite. The antioxidant system is comprised of enzymatic and non-enzymatic components which have a significant role in the prevention of stress-induced oxidative damage to key cellular macromolecules thereby impeding their functioning [17]. SOD neutralizes superoxide while H_2_O_2_ is eliminated by CAT or APX and GR [84]. Earlier increased activity of antioxidant enzymes due to exogenous treatment of trehalose has been reported in rice [85], maize [86] and sweet basil [61], thereby alleviating the oxidative damage to growth and photosynthesis. Recently, in mung bean [87] observed that trehalose application increases the functioning of antioxidants resulting in a decline in ROS accumulation, lipid peroxidation and electrolyte leakage concomitant with improved photosynthesis and yield under cadmium stress. Enzymes including APX and GR as well as AsA and GSH are among the main contributors to the ascorbate-glutathione cycle which detoxifies excess H_2_O_2_ from key cellular components including chloroplast and mitochondria thereby preventing any damage to their functioning [26, 88, 89]. Glutathione reductase maintains NADP/NADPH ratio so that electron transport does not get so much influenced [89]. Transgenic plants exhibiting up-regulated activity of antioxidant enzymes display increased growth, photosynthetic potential and photoprotection mechanisms for PSII concomitant with increased membrane stability and reduced electrolyte leakage [90]. The decline in AsA content due to Cr stress has been reported by [73] in *Ocimum tenuiflorum*. Glutathione and AsA form key non-antioxidant components regulating several aspects of plant development and metabolism in addition of their obvious role as redox components [91–93]. Glutathione protects thiol groups of proteins thereby preventing their denaturation under stress and acts as a key component for the optimal functioning of ascorbate-glutathione pathway and glyoxalase cycle [94]. Therefore, trehalose-mediated enhancement in the AsA and GSH content prevented the ill effects of Cr on key metabolic pathways and future studies may help to know exact mechanisms.

Tolerance mechanisms in trehalose-treated plants were further improved by the significant enhancement in the accumulation of osmolytes including sugars, proline and glycine betaine. Osmolytes are multifunctional molecules serving as osmoprotectants, ROS scavengers, metal chelators, stress sensors and signaling molecules [95–98]. Cr stress enhanced proline, glycine betaine and sugars however, trehalose application caused further enhancement thereby inducing better tolerance against Cr. Cr-mediated increase in osmolytes has been reported by others [4, 5, 73, 99]. Cr lead accumulation of proline was not sufficient to counter the photodestructive effects on PSII [100]. Trehalose-induced increase in osmolyte accumulation may have benefited the metabolism and growth by tissue water content maintenance, scavenging ROS and protecting enzyme functioning. Earlier trehalose mediated an increase in the accumulation of proline [101], glycine betaine [61] and sugars [101] [60]. demonstrated a significant improvement in the accumulation of glucose, sucrose, trehalose, total soluble sugars, free amino acids and proline in wheat due to the application of trehalose reflecting a significant alleviation of the damaging effects of salinity.

Moreover, total phenols increased due to the treatment of trehalose in control and Cr stress. Earlier Cr-mediated enhancement in total phenol content has been reported in *Brassica juncea* [102] and *Plantago ovata* [103]. In wheat [104], quinoa [105] and sunflower [106] have demonstrated a significant enhancement in the content of phenols due to the exogenous application of trehalose reflecting in improved stress tolerance. heavy metal treatment induces the accumulation of phenolic compounds and trehalose further increases the contents resulting in strengthening the mechanisms against the oxidative effects triggered by heavy metals. Phenolic compounds neutralize ROS to protect the cells [107] [108]. have suggested the role of phenolic compounds in the scavenging of ROS including hydrogen peroxide and superoxide ion.

### Conclusions and future prospects

In conclusion, exogenous trehalose significantly alleviated the negative effects of Cr on growth, photosynthesis, and enzyme activity. Oxidative effects of Cr were significantly alleviated by the exogenous trehalose application evident as reduced oxidative damage. Moreover, trehalose application triggered osmolyte accumulation, activated antioxidant mechanism and phenol accumulation to ameliorate the negative influence of Cr. Photosynthesis, mineral content and nitrate reductase activity were increased thereby justifying the beneficial role of trehalose in improving Cr tolerance in *Vigna radiata*. Although this study could provide a comprehensive understanding of the mechanisms through which trehalose affects *Vigna radiata* plants under Cr-stressful conditions, the molecular mechanisms remain unknown. Thus, further studies on the mechanisms of trehalose can be worthwhile to unravel the exact mechanisms involved.

## Data Availability

The datasets used and/or analysed during the current study available from the corresponding author on reasonable request.
